# Comparison of maternal milk ejection characteristics during pumping using infant-derived and 2-phase vacuum patterns

**DOI:** 10.1186/s13006-019-0237-6

**Published:** 2019-11-06

**Authors:** Hazel Gardner, Jacqueline C. Kent, Ching Tat Lai, Donna T. Geddes

**Affiliations:** School of Molecular Sciences, M310, The University of Western Australia, 35 Stirling Highway, Crawley, Western Australia 6009 Australia

**Keywords:** Lactation, Infant feeding, Breastmilk, Milk expression

## Abstract

**Background:**

Milk ejection characteristics remain consistent throughout 12 months of lactation in women who expressed breastmilk with an electric breast pump. In addition these characteristics appear to remain constant when women are breastfeeding or pumping suggesting that milk ejection is a robust physiological response. It is not known whether the stimulation of an infant at the breast in the early post partum period influences milk ejection patterns or whether this is a programmed event. However, as more data become available on the mechanisms involved in infant feeding, pumping patterns mimicking the infant more closely may provide enhanced results. The objective of this study was to compare milk ejection characteristics obtained when using a novel infant-derived pumping pattern with an established 2-phase pattern.

**Methods:**

A convenience sample of ten lactating mothers, 1 to 40 weeks of lactation with normal milk production were recruited in 2015. Each participated in two pumping sessions in which either a 2-phase pattern or infant-derived pattern were randomly assigned. Milk volume and milk ejection characteristics were recorded and the percentage of available milk removed (PAMR) was calculated. Statistical analysis used linear mixed effects modeling to determine any differences between breasts and pump patterns with the consideration of individual variability as a random effect.

**Results:**

The number of milk ejections and milk ejection characteristics did not differ between patterns. Milk volumes removed were 53.6 ± 28.5 ml (PAMR 58.2 ± 28.4) for the 2-phase pattern and and 54.2 ± 26.3 ml (PAMR 52.2 ± 22.3) for the infant derived pattern. Peak milk flow rates were positively associated with the available milk (*p* = 0.0003) and PAMR (*p* = 0.0001), as was the volume of milk removed during each milk ejection (*p* = 0.001 and *p* = 0.0001).

**Conclusion:**

An experimental pumping pattern designed to resemble infant sucking characteristics did not alter milk ejection characteristics or milk removal parameters compared with an established 2-phase pattern. Theses findings provide further evidence that milk ejection is a robust physiological response.

## Background

Human milk is a complex fluid providing the infant with optimal nutrition and immunological protection. The processes of milk synthesis and milk ejection ensure the continued provision of milk for the breastfeeding infant. Milk is synthesized by the lactocytes that line the breast alveoli where the majority of milk is stored. Milk synthesis in established lactation is regulated via autocrine control whereby continued synthesis is reliant on the removal of adequate volumes of milk from the breast [1]. The milk is made available to the infant via the milk ejection reflex during which the ejection of milk occurs as a result of sensory stimulation of the nipple by the infant, which initiates the release of oxytocin into the maternal circulation. Oxytocin subsequently binds to receptors on the myoepithelial cells that surround the alveoli causing them to contract and resulting in the expulsion of milk into the lactiferous ducts [2–4], through which it travels to the nipple and is available for removal by the infant or breast pump. Hence successful milk removal is dependent on positive pressure generated by the milk ejection within the breast and negative pressure generated by the sucking of the infant.

With the prevalence of assisted births, medications, premature birth and maternal illness along with increasing pressure for women to return to work, it is not always possible for women to feed their infant at the breast for every feed. As it is critical that milk is removed from the breast to ensure establishment and maintenance of a full milk supply [5], mothers often utilize an electric breast pump. It is imperative that pumping is efficient, effective and comfortable to achieve and maintain full lactation. The milk volume and percent available milk removed (PAMR) during breastfeeding and pumping have been shown to be greater with higher numbers of milk ejections [3, 6]. Thus, it is possible that altering milk ejection patterns may improve pumping outcomes for mothers.

This study aimed to compare maternal milk ejection characteristics during pumping with an established 2-phase pattern (Symphony) or a novel infant-derived pattern.

## Methods

### Participants

A convenience sample of ten breastfeeding mothers was recruited. All mothers had milk productions within the range reported by Kent et al. [7, 8] and were providing adequate milk to support normal growth and development of their infant (Table 1).

**Table 1:** Maternal and infant characteristics

		Mean (SD)	Range
Mother	Age (years)	34 (6)	28–45
	Parity	2 (1)	1–4
Infant	Gestational age at birth (weeks)	38 (2)	33–40
	Current age (weeks)	17 (13)	1–40
Breastfeeding	Storage capacity left breast (ml)	219 (76)	114–323
	Storage capacity right breast (ml)	181 (59)	114–303
	Number of feeds in 24 h	11 (2)	7-14
	Milk consumed from left breast (ml)	389 (160)	115–612
	Milk consumed from right breast (ml)	328 (116)	132–552
	Total volume milk consumed in 24 h (ml)	781 (139)	628–1038

### 24 hour milk productions

**Fig. 1:**
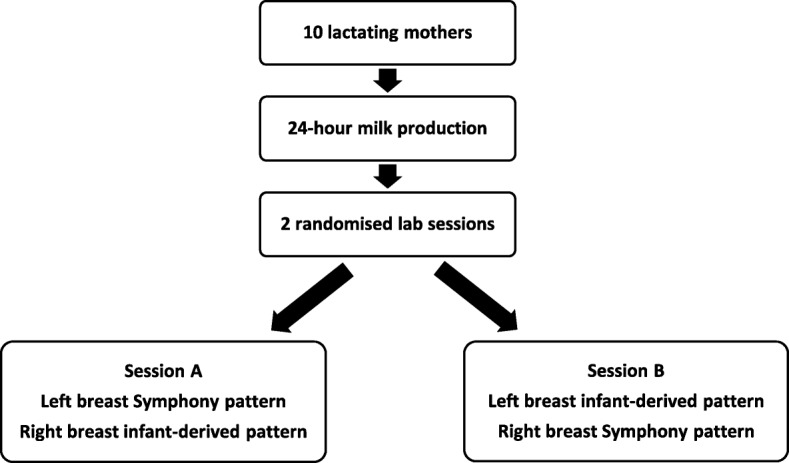
Research protocol to examine differences in milk ejection characteristics using Symphony and infant-derived pump patterns

The research protocol is illustrated in Fig. 1. Mothers measured their 24-h milk production by test-weighing at home using accurate digital scales (BabyWeigh™, Medela Inc., McHenry, IL, USA, resolution 2 g, accuracy ±0.034%). The corrected 24 h milk production was calculated using the method described by Arthur et al. [9]. During the 24-h period, mothers expressed small samples of milk into 5 mL polypropylene tubes (Sarstedt, Germany) before and after each feed which were kept frozen at − 20 °C until transferred to the laboratory for analysis. The cream content of the samples was measured using the creamatocrit method [10] which enabled estimation of breast storage capacity fullness and degree of breast fullness [11, 12]. This allows calculation of the amount of milk available in the breast at the beginning of each pumping session, and therefore the percent of available milk removed during pumping.

### Infant-derived vacuum pattern

**Fig. 2:**
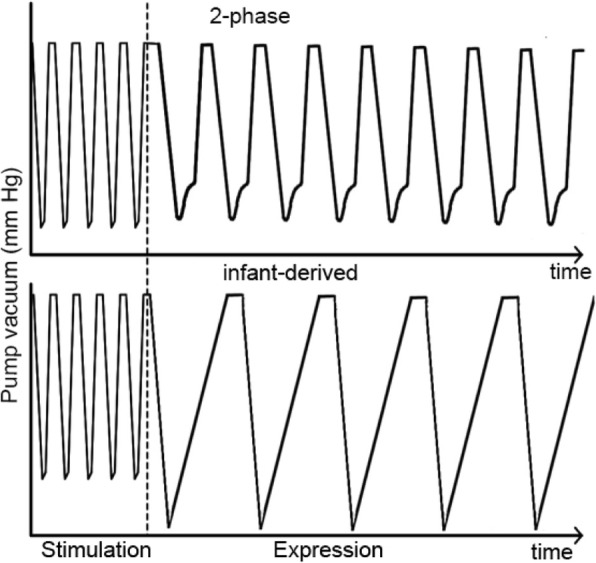
Vacuum curves for the 2-phase and infant-derived pump patterns

The first 4 min of nutritive sucking during breastfeeding of 10 fully breastfed infants were analysed for sucking frequency (sucks/min), duration of the suck and duration of beginning of vacuum application from baseline to the peak of the suck curve and the slope of the vacuum curve. A mean expression curve (the infant-derived pattern) was generated that was asymmetrical (shorter time to peak vacuum in the first half of the cycle) and operated at a frequency of 51 cycles/minute (Fig. 2). Both the infant-derived and 2-phase patterns (Symphony, Medela AG, Baar, Switzerland) share the same stimulation phase.

### Pumping sessions

The two vacuum patterns that were used were the standard Symphony pattern (Symphony, Medela, AG, Baar, Switzerland) at 54–78 cycles/minute or the infant-derived pattern. The mothers attended the research laboratory at The University of Western Australia for two study sessions. During each session, the left breast was pumped first, followed by the right breast. The vacuum pattern that was applied to the left breast during the first session was randomized, and the alternate pattern was applied to the right breast. During the second session, the two vacuum patterns were applied in the alternate order. The pump (LactaSearch, Medela, AG, Baar, Switzerland) initially applied a stimulation pattern of 120 cycles per minute (stimulation phase) until the mother sensed milk ejection or when copious milk flow began. The pump was then changed to the allocated vacuum pattern and set at each mother’s maximum comfortable vacuum, which has been shown to produce optimal milk flow rate and milk yield [12], for 10 min. The expressed milk was collected using a Showmilk (Medela AG, Zug, Switzerland) which recorded cumulative weight and milk flow rate as previously described [13]. Showmilk allowed measurement for each milk ejection: volume of milk expressed, peak milk flow rate, duration from the start to the finish, time from start of milk ejection until maximum milk flow was reached (time to peak), as previously described [14].

### Statistical analysis

Data analyses were conducted using R studio version 0.93 [15]. Data exploration consisted of descriptive statistics and box plots were constructed to illustrate the variability of the results between the two patterns. Linear mixed effects models were utilized to determine any differences between breasts and pump patterns with the consideration of individual variability as a random effect. Differences were considered statistically significant at *p* < 0.05.

## Results

Maternal and infant characteristics are documented in Table 1. Summary data of the first two milk ejections for the 2-phase and infant-derived patterns for each breast are shown in Table 2. The first two milk ejections were analysed as not all mothers had 3 or 4 ejections, limiting statistical analysis. The mean number of milk ejections for each pattern is shown in Table 2 (range 1–4).

**Table 2:** Characteristics of the first two milk ejections (ME) during pumping with either the 2-phase or infant patterns

	Overall	Left Breast	Right Breast	Symphony	Infant Pattern
	Mean (SD)	Mean (SD)	Mean (SD)	Mean (SD)	Mean (SD)
Milk Ejection 1					
Time to ME1 (min)	1.5 (1.6)	1.5 (2.0)	1.6 (1.2)	1.5 (1.4)	1.5 (1.8)
Milk volume (ml)	24.4 (16.1)	28.6 (17.7)	20.2 (13.5)	24.5 (16.1)	24.2 (16.5)
% Total milk	47.3.(24.1)	53.4 (25.7)	41.1 (21.4)	48.6 (24.1)	45.9 (24.7)
Peak Flow Rate	0.3 (0.2)	0.4 (0.2)	0.3 (0.2)	0.3 (0.2)	0.3 (0.2)
Time to peak (min)	1.0 (0.7)	1.0 (0.6)	1.1 (0.9)	1.0 (0.6)	1.1 (0.9)
Duration (min)	2.3 (0.9)	2.3 (0.9)	2.4 (0.9)	2.3 (0.9)	2.4 (0.9)
Milk Ejection 2					
Milk volume (ml)	21.9 (18.8)	17.7 (9.0)	26.0 (24.4)	21.0 (19.5)	22.9 (18.6)
% Total milk	39.4 (18.0)	36.3 (21.4)	41.0 (14.1)	38.3 (20.0)	40.7 (16.1)
Peak Flow Rate	0.3 (0.2)	0.3 (0.2)	0.4 (0.3)	0.3 (0.2)	0.3 (0.2)
Time to peak (min)	0.6 (0.3)	0.6 (0.4)	0.6 (0.3)	0.6 (0.2)	0.7 (0.4)
Duration (min)	2.2 (1.2)	2.3 (1.5)	2.1 (0.7)	2.0 (0.7)	2.4 (1.5)
Volume (ml)	53.9 (27.1)	51.9 (20.8)	55.1 (31.6)	53.6 (28.5)	54.2 (26.3)
Number of MEs	2.7 (0.8)	2.6 (1.0)	2.8 (0.7)	2.6 (0.8)	2.8 (0.9)

With respect to milk removal, neither the volume nor PAMR during milk ejections were different between breasts (*p* = 0.93, *p* = 0.65; Table 2) or between patterns (*p* = 0.39, *p* = 0.81; Table 3). Increased available milk and PAMR were associated with increased peak flow rate (available milk: *p* = 0.003; PAMR: *p* = 0.0001) and the volume of milk removed during each milk ejection (available milk: *p* = 0.001; PAMR: *p* = 0.001).

**Table 3:** Breast fullness and percent available milk removed (PAMR) overall and during each of the milk ejections (MEs)

	Infant-derived pattern		2-phase pattern	
	n	mean (SD)	n	mean (SD)
Breast fullness (proportion)	20	0.6 (0.3)	20	0.5 (0.3)
Available milk (ml)	20	109.1 (35.3)	20	107 (52.6)
PAMR Total (%)	20	52.2 (22.3)	20	58.2 (28.4)
PAMR ME1 (%)	20	23.8 (20.6)	20	25.0 (14.1)
PAMR ME2 (%)	18	21.4 (15.9)	19	24.4 (19.9)
PAMR ME3 (%)	13	10.6 (5.9)	11	13.8 (7.6)
PAMR ME4 (%)	4	5.3 (3.1)	2	10.4 (8.5)

**Fig. 3:**
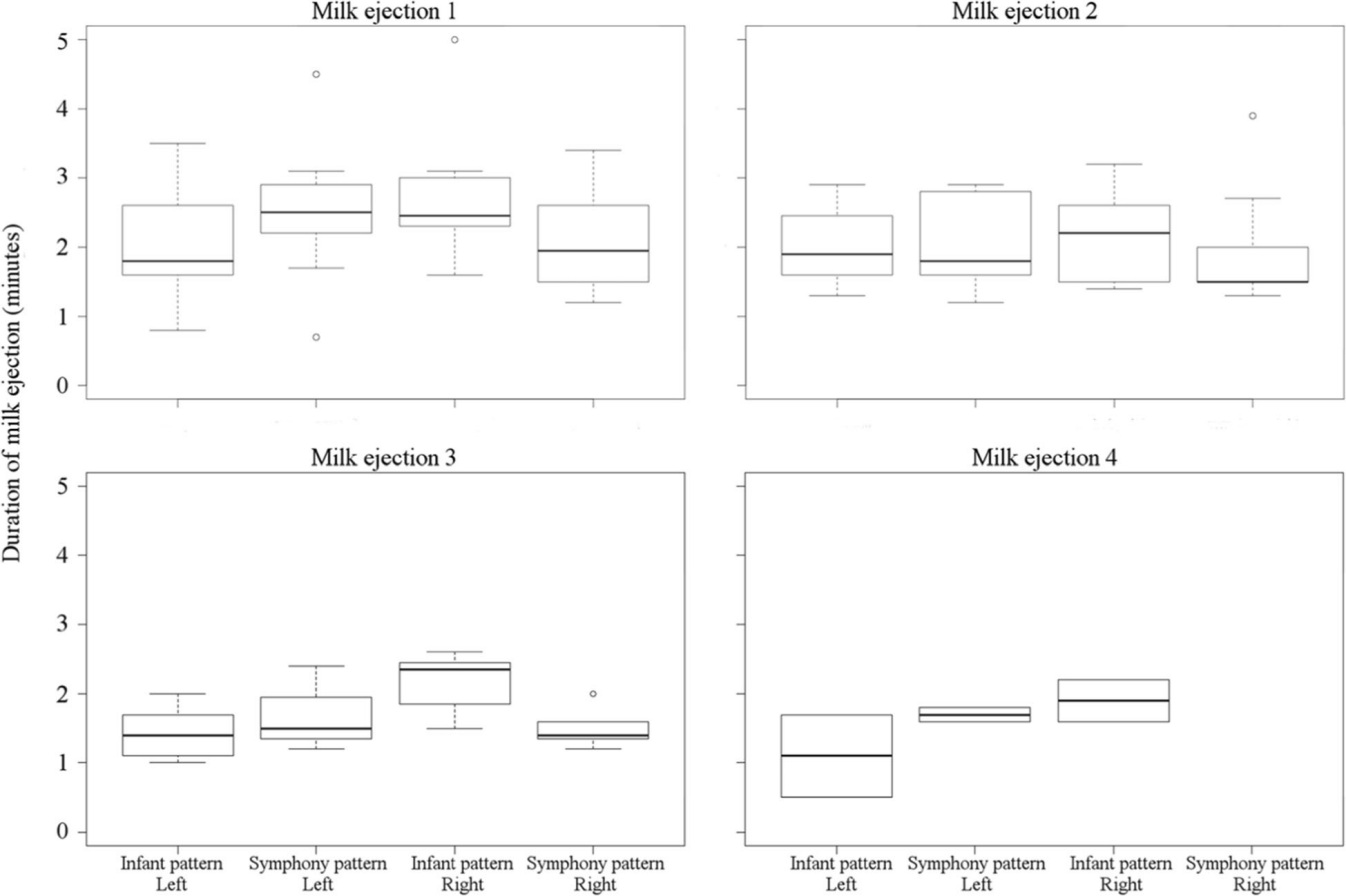
Duration of milk ejections (ME) for right and left breasts using 2-phase and infant patterns

**Fig. 4:**
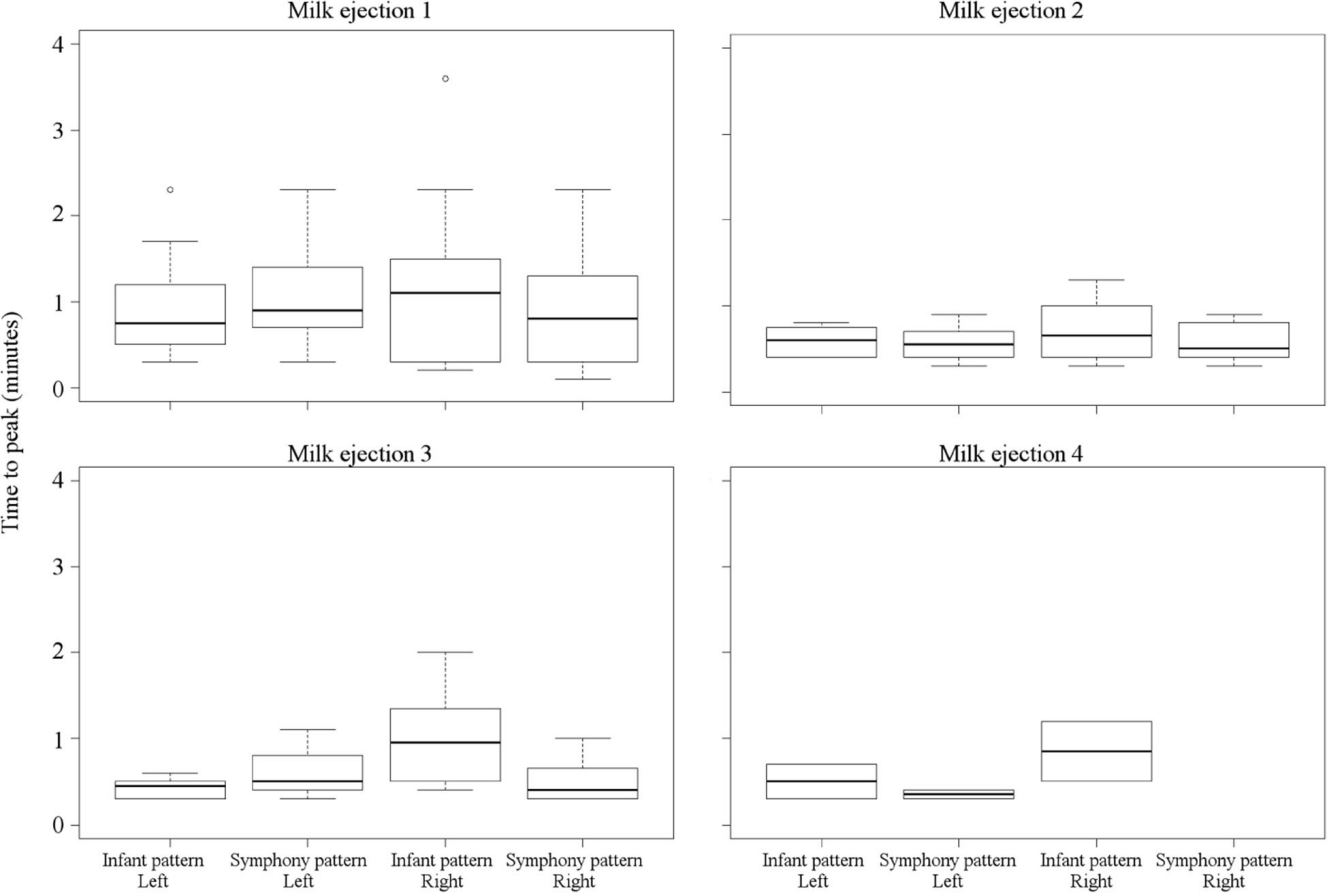
Time to peak flow rate during milk ejections using 2-phase and infant-derived patterns

The characteristics of milk ejections were consistent between breasts and between patterns with respect to duration of milk ejection (*p* = 0.94, *p* = 0.26; Fig. 3) and time to reach peak milk flow rate (*p* = 0.14, *p* = 0.15; Fig. 4), except for the time taken to reach peak milk flow rate which was longer for the first milk ejection overall (*p* = 0.001; Fig. 4).

## Discussion

In this study, we sought to determine whether a pumping pattern closer to that of infant sucking at the breast would alter milk ejection characteristics within women. We found no significant differences in either milk ejection parameters or milk removal, providing further evidence that early programming of the reflex is not influenced by different modes of stimulus later in lactation.

Milk ejection is a critical component in the continuity of milk synthesis and for the delivery of sufficient milk to the infant or effective milk removal by the pump should the mother be unable to breastfeed. It is essential that the infant consumes adequate milk as nutritional deficiencies in early life both compromise survival and preclude optimum development. Under-nourishment during this critical period may result in environmental adaptations, which manifest themselves in metabolic and other disorders later in life [16–18].

The infant is often able to remove larger volumes of the milk from the breast compared to an electric breast pump [3, 6] and fares better at maintaining milk production in the long term. Many factors likely contribute to more effective milk removal by the infant and one speculation is that there is a complex array of interactions between mothers and infants in the early postnatal period [19]. In the early stages of lactation prior to secretory activation, sucking is disorganized and characterized by a rapid sucking rate and irregular sucking rhythm [20–23], which has been thought to play a role in programming the initiation and volume of milk production [24]. A pump pattern simulating this pattern has been developed and proven to be effective in preterm mothers in establishing a greater milk supply, compared to those not exposed to the pattern [24]. In addition, this pattern decreased the time to secretory activation and increased milk output in preterm, late preterm and term mothers who were pump dependent [25], suggesting the malleability of lactation in the early post-partum period.

The role of the infant, if any, in programming or dictating the reflex has not yet been confirmed, but we have shown previously that the pattern of milk ejection, measured as duct dilation on ultrasound during breastfeeding, was similar within women to pumping with the Symphony pattern, measured by Showmilk [14].

To compare the Symphony and infant-derived patterns, we measured numerous milk ejection characteristics including the duration of milk ejection. The absence of a difference between the two patterns in duration of milk ejection indicates the infant-like stimulus did not stimulate more milk ejections or more frequent shorter milk ejections, scenarios that might improve the efficacy of milk removal. Previously we tested a 3-phase pattern where the expression pattern was changed after 2 min. This did not result in stimulation of another milk ejection and is consistent with the pump pattern itself not having an impact on milk ejection. Interestingly, the peak flow rate and time to reach peak flow rate were also not different between the two patterns (Table 2) but were similar to values reported by previous studies [14, 26]. These findings support the notion that milk ejection is an innate response unaffected by changes in stimulus [14] or strength of vacuum applied [27], and remains unaltered throughout lactation [26].

The repeatability and consistency of milk ejection patterns in women during established lactation is rather remarkable and suggests the milk ejection reflex is a robust physiological process. The ability to initiate milk ejection even without nervous stimulation (demonstrated in cases of quadriplegia [28, 29]) imply this process is critical to survival of the species. Indeed, mice pups of oxytocin knockout dams die soon after birth due to the lack of milk transfer [30].

Healthy infants who breastfeed effectively are often thought to be more efficient than the expression of milk either by hand or with an electric breast pump. Breastfed infants have been shown to remove 50% of the total volume of milk removed at a breastfeed in the first 2 min and 80% in 4 min [31]. Pumping with the Symphony pattern has been shown repeatedly to remove 80% of the total volume of milk pumped in the first 8 min of a 15 min pumping session [6] and that it removes 50 to 75% of the available milk in the breast [12, 13]. The PAMR for both patterns in this study (Table 3) was lower than those reported previously. However, the pumping sessions in this study were 10 min in duration compared to 15 min in previous studies [12, 13].

When exploring the dynamics of milk removal from the breast during pumping it has been shown that the majority of the milk (76% on average) is removed during the first two milk ejections [12]. In this study 90% of the total milk pumped was removed in the first two milk ejections (Table 2). However, this higher percentage may be due to the shorter pumping time and resultant lower volume. In contrast, the PAMR for milk ejections would be a better indicator of the effectiveness of milk removal. PAMR for the first milk ejection was between 24 and 28% on average which is similar to that found by Ramsay et al. [32] who used the Symphony pattern. The first two milk ejections in this study removed 45 to 49% of the milk available whereas the infant removes on average 70% of the available milk during a breastfeed [7]. If 80% of the milk is removed by the infant in the first 4 min of a feed, equivalent to the first two milk ejections, then the pump is still on average less efficient whether or not the Symphony or infant-derived pattern is used [31].

As the breast empties, the rate of milk flow changes during subsequent milk ejections, suggesting that infants modify their sucking patterns to accommodate these changes in flow [12, 13, 33]. Cannon et al. showed that infants modify their sucking characteristics between the first and second two-minute nutritive phases of a feed [34]. In particular, the infant oral vacuum is reduced in strength between the first two-minute phase and subsequent phases during the feed, which could be a response to changes in milk flow or due to the increasing satiety of the infant [34]. Characterising milk ejection during pumping to allow manipulation of vacuum strengths or patterns may optimize milk removal, although a change in expression pattern at 2 min to a more effective pattern later in pumping failed to achieve this [32]. Interestingly, peak flow rates for the first two milk ejections in this study were not different irrespective of the pattern, which may be a reflection of the similar degrees of fullness of the breast which are positively related to milk flow rate [26].

One of the strengths of this study was that one of the pump patterns was developed from breastfeeding data to attempt to emulate infant sucking. This exploratory study had a relatively small sample size however the results of consistency of milk ejection patterns support previously published results. Investigation of milk ejection patterns in the immediate postpartum period would be useful to confirm that milk ejection patterns are programmed rather than developed or altered at birth.

The characterisation of milk ejection in individuals may have other advantages; potentially reducing pumping time in mothers in whom most milk is removed within the first few minutes while those that release milk later in the pumping period may need to pump for longer. Further exploration of alternative pumping patterns would also be worthwhile to improve efficacy for mothers that are pump dependent.

## Conclusion

An infant-derived pumping vacuum pattern did not alter maternal milk ejection characteristics when compared with the 2-phase Symphony pump pattern, providing further evidence that milk ejection is a robust physiological response.

## Data Availability

The datasets generated during and/or analysed during the current study are available from the corresponding author on reasonable request.
